# The History of Medicine

**Published:** 1895-03

**Authors:** 


### The History of Medicine. By Roswell Park, M. D., Professor of Surgery, University of Buffalo.

This embraces not alone the history of medicine, its evolution and progress from the earliest times down to the present, but likewise deals with mythological factors, and the political, religious, and philosophical issues of the science.

Delivered in Alumni Hall, Medical Department of the University of Buffalo, during October, November, and December of 1894, these lectures will now appear, *exclusively*, in the *Medical Age*, beginning with the issue of January 10th, 1895.

Also will appear during the year special dissertations upon Malaria, Remittent and Typhoid Fevers, by Prof. Joao Vincente Torres Homem; Fevers of Infancy and Childhood; and Reminiscences of a Medical Naval Officer Abroad.

*The Medical Age*, semi-monthly, $1.00 per year. P. O. Box 470, Detroit, Mich.

